# Women’s experiences of navigating gestational diabetes in Denmark: a qualitative study of healthcare and self-care, with a focus on dietary education and delivery

**DOI:** 10.1186/s12884-026-09055-8

**Published:** 2026-04-16

**Authors:** Bettina Ewers, Emma Davidsen, Marianne Juhl Hansen, Karoline Kragelund Nielsen

**Affiliations:** 1 Department of Diabetes Care, Steno Diabetes Center Copenhagen, Borgmester Ib Juuls Vej 83, Herlev, 2730 Denmark; 2 Department of Clinical and Translational Research, Copenhagen University Hospital - Steno Diabetes Center Copenhagen, Herlev, Denmark; 3 Department of Prevention, Health Promotion Research & Community Care, Copenhagen University Hospital – Steno Diabetes Center Copenhagen, Herlev, Denmark

**Keywords:** Dietary education, Dietary delivery, Gestational diabetes mellitus, GDM, Healthcare, Healthcare support, Selfcare, Physical activity, Qualitative study

## Abstract

**Background:**

Gestational diabetes mellitus (GDM) requires women to rapidly integrate new dietary self-care practices into everyday life during pregnancy. Dietary education is a central component of GDM management, and in Denmark it is often offered as a single dietitian session, either through individual counselling or group-based education. However, limited attention has been given to how women experience dietary education and their preferences regarding its timing, format, and continuity, and how these features influence their ability to engage in dietary self-care. This study explores women’s experiences with healthcare and self-care in GDM within a Danish context, focusing on dietary education and how its delivery influences women’s ability to manage their condition.

**Methods:**

Semi-structured qualitative interviews were conducted with 20 women with GDM. Data were analysed abductively using Braun and Clarke’s reflexive thematic analysis framework.

**Results:**

Six themes were identified: (1) timely information, (2) information sources, (3) support from healthcare professionals, (4) group education delivery, (5) unmet self-care needs, and (6) dietary challenges during the transition to insulin therapy. Women described the period following GDM diagnosis as particularly challenging, highlighting unmet needs for timely, concrete, and actionable dietary guidance. Many relied on informal networks, including family, friends, and online communities, to supplement formal healthcare information. While peer interaction was valued by some, group-based dietary education was often perceived as insufficiently tailored to individual needs. Inconsistencies in dietary advice across healthcare professionals, limited opportunities for dietary follow-up, and insufficient support during the transition to insulin therapy contributed to uncertainty and frustration in dietary self-care.

**Conclusions:**

The study identifies important gaps in the delivery of dietary education for women with GDM in Denmark, extending existing knowledge by highlighting how timing, format, continuity, and interdisciplinary coordination shape self-care experiences. These findings underscore the need for earlier, more individualised, and better-coordinated dietary education, including support during key transition points such as insulin initiation. Addressing modifiable features of healthcare delivery may enhance self-care, reduce uncertainty, and improve health-related outcomes for women with GDM and their offspring.

**Supplementary Information:**

The online version contains supplementary material available at 10.1186/s12884-026-09055-8.

## Background

Gestational diabetes mellitus (GDM) is a transient condition characterised by glucose intolerance and hyperglycaemia that arises or is first diagnosed during pregnancy, most commonly in the second trimester [1]. Inadequate glycaemic management is associated with adverse short-term maternal and neonatal outcomes, including foetal overgrowth, preterm birth, preeclampsia, neonatal hypoglycaemia, and admission to neonatal intensive care [2–4]. Although GDM typically resolves after delivery, hyperglycaemia in pregnancy is also associated with higher risk of obesity, type 2 diabetes, and cardiovascular disease for both mother and child in later life [5, 6]. Consequently, effective education and engagement in self-care to achieve recommended glycaemic targets during pregnancy are critical to reducing both short- and long-term complications.

According to clinical guidelines, first-line treatment following a diagnosis of GDM includes self-care education and support, encompassing most importantly dietary modification guided by a dietitian, but also self-monitoring of blood glucose (BG), and engagement in physical activity to support adequate nutrient intake, appropriate gestational weight gain, and achievement of glycaemic targets during pregnancy [7, 8]. These recommendations are supported by evidence from systematic reviews and meta-analyses demonstrating that dietary modifications improve maternal glycaemic control, reduce the need for insulin therapy, and lower offspring birth weight among women with GDM [9, 10]. Danish cohort data further indicate that adherence to diabetes-specific recommendations, in combination with lower gestational weight gain, is associated with improved glycaemic outcomes and reduced foetal growth [11]. Evidence regarding the overall effects of physical activity on pregnancy outcomes in GDM remains limited; however, light- to moderate-intensity postprandial activity has been shown to temporarily reduce BG levels in a dose-dependent manner [12–14]. For approximately one-third of Danish women with GDM, behavioural changes alone are insufficient to meet recommended glycaemic targets, necessitating insulin therapy during pregnancy [11, 15], a proportion comparable to international reports [16–18].

Despite the recognised importance of self-care education and support in the management of GDM, evidence guiding the optimal mode of educational delivery remains limited, both for women managed with dietary modification alone and for those requiring insulin therapy. Based on empirical mapping across Danish hospital dietetic services (unpublished data collected in 2023), routine hospital-based care following a GDM diagnosis typically includes written educational materials, hands-on instruction in BG monitoring, and referral to a dietitian. Dietary education is most often provided as a single session, delivered either individually or in a group, and offered in person or virtually depending on local hospital organisation, available resources, and language considerations. Routine follow-up dietetic support is uncommon. Limited evidence on the effectiveness of different educational delivery approaches—both in terms of clinical outcomes and women’s perceived support—may contribute to the observed variation in standard practice. Similar heterogeneity in dietetic care for GDM has been reported internationally, with no universally agreed standard regarding the organisation and delivery of dietary education [19].

The need to rapidly understand and implement complex self-care recommendations following a GDM diagnosis can be emotionally demanding and challenging to navigate within healthcare settings. Accordingly, emotional distress related to GDM self-care has been reported among women across treatment modalities, including both diet- and insulin-treated women [20–22]. Health literacy—the ability to access, understand, and use health information [23]—has also been found to influence adherence to self-care recommendations and glycaemic control in diabetes [24, 25]. In this context, health literacy-directed educational interventions have been associated with more favourable health outcomes among women with GDM [26, 27]. However, while previous research has highlighted health literacy challenges among certain subgroups, including immigrant women [28–30], this emphasis may obscure broader determinants of engagement with diabetes care. Moreover, differences in preferences for self-care and dietary education delivery may be relevant even among women with adequate language skills and access to healthcare services.

The present study aimed to explore women’s experiences of healthcare and self-care following a diagnosis of GDM in Denmark. The study was conducted within a clinical context where dietary education was primarily delivered through group-based sessions for Danish-speaking women and individual dietary counselling for non-Danish-speaking women. With a specific focus on dietary education and its delivery, the study aimed to identify unmet needs and opportunities for improvement in self-care education. Furthermore, we aimed to explore potential differences in experiences and support needs among a diverse group of women with GDM, including women managed with dietary modification alone or insulin therapy, and women of both Danish and non-Danish origin.

## Methods

### Study design

The study followed a qualitative research design using semi-structured, in-depth interviews to explore the women’s perceptions and experiences (see Sect. “Interview guide”).

### Study setting

In Denmark, GDM is diagnosed using a selective screening procedure [31]. Women undergo early screening with a 75-g oral glucose tolerance test (OGTT) between gestational weeks 10 and 20 if they have a history of GDM or present with two or more risk factors, including a prepregnancy body mass index (BMI) ≥ 27 kg/m^2^, a family history of diabetes, previous delivery of a baby weighing ≥ 4500 g, polycystic ovarian syndrome (PCOS) or glucosuria [31]. Screening is also performed between gestational weeks 24 and 28 in women with one risk factor or in those with twin pregnancies. According to Danish guidelines, GDM is diagnosed if the 2-h plasma glucose concentration following OGTT is ≥ 9.0 mmol/L [31]. Shortly after diagnosis, women are instructed to monitor their BG levels six times daily, with BG target ranges specified for pre-meal and postprandial values [8]. At the local hospital, dietary education was recommended shortly after diagnosis, preferably within the first week. Danish-speaking women were offered one group-based session, whereas non–Danish-speaking women were offered one individual dietary counselling session, either in person or by telephone. After approximately 7–14 days on the dietary regimen, women were reviewed by an obstetrician to assess whether BG targets had been achieved. According to national Danish guidelines, if more than two BG measurements within a week exceed target levels without an identifiable explanation, insulin therapy is initiated, and women continue BG monitoring six times daily until delivery [8]. Among diet-treated women who achieve BG targets, monitoring frequency may be reduced to 2–3 days per week until delivery [8].

### Recruitment

Participants were recruited from a department of obstetrics and gynaecology at a major hospital in the Copenhagen area, Denmark. Women were eligible for participation if they had a current GDM diagnosis and were receiving treatment for GDM at the recruiting hospital. Exclusion criteria included limited proficiency in Danish or English, severe pregnancy complications, or increased mental vulnerability as assessed by an obstetrician. Language proficiency criteria reflected pragmatic considerations aimed at facilitating participation and minimising participant burden, as interviews were conducted either immediately following obstetric consultations or as online interviews without interpreter support, requiring participants to engage directly in the interview. Purposeful sampling was used to ensure representation of the diverse characteristics of women with GDM within the Danish population. Sampling aimed to capture variation in treatment regimen (diet only or insulin therapy), as well as prior GDM diagnosis, parity, and ethnic background [32]. The sampling strategy targeted that approximately half of the participants were insulin-treated, and that half were of non-Danish ethnic origin. Initial recruitment was facilitated by two obstetrician specialists in GDM during routine consultations. Women interested in participating received an information letter, available in Danish or English, detailing the study’s objectives, the use of pseudo-anonymity, and the voluntary nature of participation. The time and location of the interview were determined in agreement between the women accepting to participate and the interviewer (BE or MJH). Written informed consent was obtained before the interviews, including consent for audio recording and the use and presentation of anonymised self-reported personal data. The sample size was determined using the concept of data saturation, defined as the point at which no new information or analytically generated themes were identified in the data [33, 34].

### Participants

A total of 25 eligible women were invited to participate, all of whom agreed to be contacted. Of these, three could not be reached, and two declined due to lack of time and energy to participate. Data collection was finalised after conducting interviews with 20 women, as data saturation was considered reached. The interviews were conducted at various stages of pregnancy following a GDM diagnosis. Twelve participants were interviewed during late pregnancy, between gestational weeks 35 and 40. The rest were interviewed earlier between weeks 19 and 34, with the earliest interviews conducted at weeks 19–20.

### Data collection

Between November and December 2022, interviews were conducted by two experienced dietitians specialising in diabetes care and research. Notably, neither of these dietitians was involved in the clinical management of GDM at the time of the study, as dietary therapy for GDM was provided by the hospital-based dietitians at the study site. Both interviewers were cisgender women in their 40 s of Danish descent and had been pregnant and given birth but had no personal history of GDM.

Depending on participants’ preferences, interviews were conducted in person (*n* = 10), online via video (*n* = 9), or over the telephone (*n* = 1). Four interviews were conducted in English and the remainder in Danish. In four interviews, the women’s partners were present. Participants were informed that BE was the project leader and MJH a project worker in the study; their educational backgrounds were not disclosed to minimise potential response bias. BE and MJH had no prior relationship with the participants. However, the interviewers’ professional backgrounds in diabetes care may have influenced the interview process, including their sensitivity to clinical practices and self-care challenges in diabetes. This was addressed using an open-ended interview guide and an emphasis on participants’ own perspectives.

All interviews were audio-recorded, and no repeat interviews were conducted. The interviews lasted between 32 and 66 min. Before each interview, participants were informed that the study aimed to explore their experiences of managing GDM, with particularly emphasis on dietary education and its delivery. The interviews were designed to generate insights into women’s interactions with healthcare services, access to information, support needs, and preferences related to delivery of self-care education, to inform the development and testing of a self-care education programme for GDM, with the potential to improve future GDM care and support. Participants were assured that there were no right or wrong answers and that they could decline to respond or provide minimal responses if they preferred.

#### Interview guide

The interviews were conducted using a semi-structured interview guide with predefined topics and questions (supplementary file 1), providing a flexible framework for discussion. Selected open-ended questions in the interview guide were inspired by the Conversational Health Literacy Assessment Tool (CHAT), a tool for identifying strengths and barriers related to health literacy [35–37]. These questions were used to explore topics such as supportive personal relationships, interactions with healthcare professionals, access to and comprehension of health information, and current health behaviours. The interview guide covered eight main topic areas: (1) initial reactions to the diagnosis; (2) understanding and personal experiences of the condition; (3) social disclosure and family and other social support following the diagnosis; (4) managing everyday life with GDM; (5) experiences with healthcare services following diagnosis; (6) health knowledge and sources of information; (7) perceptions of health and related self-care behaviours; and (8) perceived needs and preferences for optimising GDM care and support. The guide was iteratively developed and refined through discussions with experienced qualitative health researchers. The participants were asked about the adequacy of self-care education and support they received, as well as additional services or resources they found beneficial for improving and maintaining healthier behaviours during pregnancy to better manage GDM. They were also encouraged to identify educational support and tools that could help future women with GDM. To facilitate this, an overview of educational and supportive activities was provided, featuring images of various group-based education, individual dietary counselling, digital platforms, virtual consultations, wearables, exercise classes, and one-on-one physiotherapy sessions.

### Data analysis

Data were analysed using reflexive thematic analysis following Braun and Clarke’s framework [38–40]. An abductive analytic approach was adopted, combining inductive, data-driven coding with theory-informed interpretation. In contrast to a purely inductive approach, which derives themes solely from the data, or a deductive approach, which applies predefined theoretical categories, the abductive approach involved an iterative movement between empirical data and theoretical perspectives, allowing emerging patterns to be explored and refined through relevant conceptual lenses. Interviews were transcribed verbatim by two research assistants and checked for accuracy against the audio recordings. During the initial phase, authors BE and ED independently read five transcripts and discussed emerging patterns in relation to the study aim and theoretical framework. BE subsequently coded all transcripts using NVivo software (version 14, Lumivero, Melbourne, Australia). Codes were iteratively grouped into themes and sub-themes through ongoing interpretation and regular discussions among the author group (ED, MJH, and KKN), ensuring that themes were coherent, distinct, and grounded in the data [38, 41]. Analysis continued throughout the writing process, culminating in the final themes presented. All illustrative quotes were translated into English and confirmed by co-authors to ensure accuracy and consistency with the original meaning.

The author team comprised clinical and public health researchers with expertise in diabetes care, self-care educational interventions, and GDM research, including stigma, migration, and family-based interventions following delivery. These professional backgrounds may have influenced analytical interpretations and expectations regarding healthcare and self-care needs in GDM. To address potential biases and strengthen analytic rigour, we adopted a reflective and transparent approach, including regular discussions throughout the analytic process to critically examine how authors’ professional roles, prior knowledge, and assumptions might shape interpretations.

### Patients and the public involvement

Women with GDM from the local hospital were consulted during the early stages of the study to inform the refinement of the interview guide. This input aimed to enhance the relevance and clarity of the interview topics from a patient perspective. Patients and the public were not involved in the study design, recruitment, data collection, analysis, or dissemination.

### Ethics considerations

According to the Danish National Committee on Health Research Ethics (reference number H-22058261), qualitative health research such as this study does not require ethical approval from the Regional Ethics Committee of the Capital Region of Denmark. The study adheres to the Consolidated Criteria for Reporting Qualitative Research (COREQ) [42].

## Results

Background characteristics of the participants are summarized in Table 1. All women were cohabiting with a partner. Twelve women had given birth to more than one child, six had a prior diagnosis of GDM, ten were from ethnic minority backgrounds, and ten managed their condition through dietary measures alone, while the remaining participants required insulin therapy.

**Table 1:** Background characteristics of participants (*n* = 20)

Characteristics	***n (%) or mean (range)***
Age, years	33.8 (23.0–41.0)
Highest level of education	
Primary school	5 (25)
Vocational degree	3 (15)
Short further (≤ 2 years)	2 (10)
Medium further (3–4 years)	5 (25)
Long further (≥ 5 years)	5 (25)
Employment	
Employed	15 (75)
Unemployed	3 (15)
Student	2 (10)
Self-reported ethnic origin	
Europe^1^	10 (50)
South Asia^2^	6 (30)
Middle East and North Africa^3^	4 (20)
Cohabitating with partner	
Yes	20 (100)
Blended family	
Yes	2 (10)
Multiparity	
Yes	12 (60)
Previous GDM diagnosis	
Yes	6 (30)
Gestational age at interview, weeks	33 (19–40)
Interview language	
Danish	16 (80)
English	4 (20)
GDM management in current pregnancy	
Diet only	10 (50)
Diet + insulin	10 (50)

Data are summarized as means with ranges in parentheses or as numbers with percentages in parentheses for categorical variables

*Abbreviation*: *GDM* gestational diabetes mellitus

^1^Denmark, Bosnia, Sweden (*n* = 8)

^2^Afghanistan, Bangladesh, India, Nepal, Pakistan

^3^Iraq, Turkey, Somalia, Morocco

Six themes were identified: (1) timely information, (2) information sources, (3) support from healthcare professionals, (4) group education delivery, (5) unmet self-care needs, and (6) dietary challenges during transition to insulin therapy. The themes are presented in Table 2 with each theme followed by main findings.

**Table 2:** Six key themes and description of main findings

Theme 1: Timely information
1a. Too generic or insufficiently tailored information at diagnoses
1b. Delays in accessing specialised dietary information and guidance
Theme 2: Information sources
2a. Limited reliance on formal information sources
2b. Greater use of informal networks for information and guidance
Theme 3: Support from healthcare professionals
3a. High satisfaction with healthcare professionals’ support
3b. Ongoing dietary support from dietitians needed
Theme 4: Group education delivery
4a. Mixed experiences and attitudes towards group education
4b. Missed opportunities for experience sharing and peer interaction
Theme 5: Unmet self-care needs
5a. Need for more personalised diabetes-specific dietary education and guidance
5b. Need for clearer guidance on safe physical activity in GDM
Theme 6: Dietary challenges during transition to insulin therapy
6a. Need for knowledge and skills in carbohydrate management before transitioning to insulin
6b. Need for enhanced interdisciplinary collaboration and consistent dietary recommendations

### Theme 1 timely information

Most participants highlighted the importance of timely information after receiving their diagnosis, along with addressing common misconceptions about GDM—particularly the belief that GDM is caused by excessive sugar consumption during pregnancy. Many expressed initial feelings of guilt or self-blame, which they felt were not adequately addressed by the information provided. While written materials, particularly those delivered digitally, were intended to offer clarity and reassurance, they were often perceived as too generic or insufficiently tailored to individual concerns. For some, the lack of immediate follow-up during the initial stages of diagnosis left them feeling uncertain and inadequately informed, making it difficult to navigate dietary adjustments and BG management with confidence:

*“I think what I would have appreciated the most was if the message in my e-Boks [secure digital mailbox used in Denmark for official communication] about having diabetes in pregnancy had been followed up sooner. […] Of course, there were plenty of attachments explaining what it meant and what would happen. It was clearly stated that I’d have to monitor my blood sugar and that I’d be referred to a dietitian. […] It might have been nice if, very soon after receiving that message, there had been an opportunity to talk to someone individually about what it means for you.”* (Participant 20, Danish origin, 36 years, diet-treated, first-time GDM)

All participants expressed strong motivation to implement dietary changes immediately after their diagnosis. However, delays in accessing specialised dietary information and guidance from a dietitian were a common frustration, often hindering their ability to adopt the necessary adjustments promptly. This sense of being unprepared and unsupported was shared by women with both first-time and recurrent GDM, pointing to the absence of clear, immediate dietary information and guidance:

*“The nurse gave me a sheet with information about what to avoid and what I could eat […] and then she said, ‘just roughly follow this until you see the dietitian.’ I remember that my appointment with the dietitian got postponed […]. It ended up being three weeks after the original date. By that point, I felt frustrated—I wanted to start eating the way I should and not have to wait even longer.”* (Participant 10, North African origin, 32 years, diet-treated, first-time GDM)

### Theme 2 information sources

Delayed referrals to healthcare professionals following the GDM diagnosis often compelled women to rely on informal sources of information to understand and manage their condition. Participants had varied knowledge needs and differing capacities to seek additional information, frequently turning to personal networks—such as friends, family, peers, and online communities—for initial information. When asked about sources of information, one woman said:

*“[…] It’s especially friends, family, and my network, or other pregnant women I know. […] And then there’s just a wide range of places, like a Facebook group for people who are due in the same month. I don’t use it actively myself, but I’m a member and can see that it’s a place where you could ask a lot of questions every day that people write about”* (Participant 20, Danish origin, 36 years, diet-treated, first-time GDM)

In addition, many women sought information from the websites of Danish health authorities or professional societies. However, these websites were often perceived as lacking practical and engaging dietary guidance:

*“It would be great if, when you have gestational diabetes, there was a website from dietitians where you could see like what vegetables, fruits, or foods are good to eat. […]. Everything in one place, where you have an overview of it all. Good foods that isn’t boring, with recipes and so on.”* (Participant 5, South Asian origin, 26 years, insulin-treated, recurrent GDM)

Nevertheless, the lack of tailored, practical, and engaging dietary information and recommendations prompted some women to seek out specific dietary resources, such as YouTube channels for low-carb, high-protein recipes, or culturally tailored diets, including Indian vegetarian cuisine. Some had also found apps to use for tracking carbohydrate intake and explore healthy meal planning ideas.

Some women acknowledged that using online sources during the waiting period between diagnosis and their first consultation entailed an emotional risk:

*“[…] When you’re unsure about something, you ask the internet or Google, and you end up on sites where you can read about it. […] It makes you nervous. […] Everyone knows they shouldn’t do it, but everyone does it anyway.”* (Participant 3, Danish origin, 31 years, diet-treated, first-time GDM)

A subset of women therefore refrained from independent information-seeking entirely, relying exclusively on advice from healthcare professionals or family members with health expertise for guidance:

*“I mostly just listen to what the healthcare professionals say and advise me to do. […] It’s probably better than reading about it elsewhere.”* (Participant 4, Danish origin, 28 years, diet-treated, first-time GDM)

### Theme 3 support from healthcare professionals

Despite challenges in accessing timely professional support, women reported high levels of satisfaction with the care provided by healthcare professionals from the time of diagnosis and throughout pregnancy. They valued the time, attention, and expertise offered, particularly by obstetricians and endocrinologists. Most women felt their health concerns were adequately addressed, and communication was generally perceived as effective. They described feeling taken seriously, supported, and confident that their condition was being well-managed. Some women took a proactive approach by preparing for consultations with written questions to ensure their concerns were addressed:

*“Many questions can pop up while you’re at home and before you have your appointment, and you really want answers to them. I’ve been very focused on writing down my questions so that I could get answers. […] This has been very important for me.”* (Participant 3, Danish origin, 31 years, diet-treated, first-time GDM)

Nevertheless, several women highlighted a lack of time for emotional support during healthcare interactions:

*“It’s been very much like, ‘What’s important to eat? How does your blood sugar look? Now you need to do this, and now we’ll do this.’ It hasn’t really been, ‘How are you feeling about it?’. […] I talk about that with my family.”* (Participant 6, Danish origin, 33 years, insulin-treated, first-time GDM)

The women expressed a need for earlier dietary support from a dietitian. When this need for support could not be met e.g., due to delayed appointments with the dietitian, some participants reported receiving practical guidance on dietary choices and BG management from doctors instead:

*“If I had known I’d have a dietitian appointment in four weeks, I might have written down questions to get a more detailed explanation. […] But I’ve used my diabetes doctor for questions, if needed.”* (Participant 19, Danish origin, 41 years, insulin-treated, recurrent GDM)

The need for ongoing dietary support from dietitians was particularly evident during vulnerable periods such as stabilising BG levels, adjusting to dietary changes, or managing new challenges (e.g., eating out, travelling, or initiating insulin use). Regular follow-ups with dietitians—offered as needed and preferably virtually to minimize the burden of additional consultations—were regarded as essential for reducing uncertainty and supporting the ability to follow dietary recommendations:

*“I think I had a few periods in the beginning, where no matter what I was eating my sugar was high and it was disappointing. […] It would have been nice to have some support in that period like ‘just try this and that and change this’. […] During that period when people are trying and learning, they need more help. […] Being able to just make a call or have a video chat with a person showing what you’ve been doing and what is the problem and what is your reasons behind why your sugar is high.”* (Participant 7, South Asian origin, 37 years, diet-treated, recurrent GDM)

*“[…] Something where they called a few weeks later, after you had the consultation, to ask, ‘What worked for you?’ This worked for me, that didn’t. ‘Well, then try doing this instead.’ So, it would be great to have a follow-up consultation with a dietitian, because I feel like dietitians are the ones who really know what you should be eating.”* (Participant 5, South Asian origin, 26 years, insulin-treated, recurrent GDM)

Some participants also highlighted that follow-ups with a dietitian could be key in maintaining motivation and sustaining improved dietary habits during pregnancy:

“*Scheduling a few more dietitian consultations – some more dietary guidance […] might have helped keep me on track.”* (Participant 19, Danish origin, 41 years, insulin-treated, recurrent GDM)

### Theme 4 group education delivery

Most participants had attended a dietitian-led group education session, which was part of standard care for women with GDM at the local hospital. In contrast, non-Danish speaking women primarily received individual counselling with a dietitian, unless an English-speaking group could be assembled within a short time frame. Experiences with the group format were mixed. Most women felt that the group education session was impersonal and lacked sufficient time for optimal engagement and experience sharing. As one participant explained:

*“I think it was quite ‘smart’ to keep it general, and the group education only lasted half an hour since there weren’t many questions. […] But the group setting could have been used more effectively. […] Time could have been saved if participants had shared useful advice or personal strategies.”* (Participant 9, Danish origin, 36 years, insulin-treated, first-time GDM)

Others emphasized the emotional aspects of GDM and the missed opportunity to foster peer support for those going through similar experiences:

*“I had hoped there would be time to talk with each other […] but there wasn’t any dialogue between us. […] Everyone experiences gestational diabetes differently, and being with others in a similar situation could have been used for sharing.”* (Participant 13, South Asian origin, 32 years, diet-treated, first-time GDM)

Some women found it challenging to identify with or relate to others in a mixed group of women with GDM, particularly when they lacked shared challenges such as weight concerns or extreme glucose fluctuations:

*“I think I found it a bit difficult to identify with the others because I’ve never had issues with my weight or anything like that. […] I think I felt a bit like I had ended up in the wrong group*.” (Participant 20, Danish origin, 36 years, diet-treated, first-time GDM)

Many expressed a desire for more individualised attention tailored to their specific needs—at the very least, an individual follow-up appointment with a dietitian if the initial education was delivered in a group setting—to better address their unique circumstances:

*“I guess I kind of miss having some follow-up on it [the dietary group education]. […] Instead of just having a group come in and receive something standard, as individual needs can vary greatly.”* (Participant 13, South Asian origin, 32 years, diet-treated, first-time GDM)

When asked about education, tools and support needs related to physical activity, many participants suggested supervised group exercises to build social networks and foster peer support. This activity was viewed as an opportunity for mutual motivation, experience sharing, and creating a sense of connection. However, several barriers were also identified, including the frequent medical appointments required for GDM management, the short time frame between diagnosis and delivery, differing gestational stages among potential participants, physical limitations, and time constraints related to transportation.

### Theme 5 unmet self-care needs

Various unmet self-care needs were identified. Regarding dietary education and guidance most participants noted that the dietary education was overly generalised, offering only basic public guidelines without specific recommendations for meal planning or portion control. For instance, one participant commented:

*“There was a lack of guidance on how much to eat. […] The dietitian said, ‘Oh, you can just read about it in the pamphlet.’. […] Sort of like the ‘food pyramid’, where you just look at what foods are considered good to eat.”* (Participant 2, Danish origin, 33 years, insulin-treated, first-time GDM)

This comment reflected a broader concern: instead of diabetes-specific, personalised guidance, most participants felt they received generic recommendations that failed to address practical self-care needs in diabetes. For some, these generic recommendations merely confirmed their pre-existing knowledge of healthy eating. As one participant stated:

*“It would have probably been harder for me if I hadn’t taken the nutrition advisor course. I knew what it meant when they said to cut down on sugar and follow the dietary guidelines.”* (Participant 3, Danish origin, 31 years, diet-treated, first-time GDM)

Some women with previous GDM experience recalled receiving more diabetes-specific dietary advice at other hospitals, highlighting variations in dietary management approaches:

*“Yes, well, I’ve been on many diets over the years, so you do know how you’re supposed to eat and that was primarily what we talked about [during this pregnancy with GDM]. Whereas I feel that in previous pregnancies with GDM, the dietary advice was more diabetes-focused.”* (Participant 19, Danish origin, 41 years, insulin-treated, recurrent GDM)

Additionally, participants expressed a strong desire for practical education on carbohydrate management, including clear guidelines on what to include or avoid. They emphasized the need for tools such as detailed meal plans, snack suggestions, recipes, and visual aids illustrating recommended carbohydrate amounts:

*“I was completely confused about how to eat properly, and it would have been nice to have examples of what to eat for breakfast, lunch or snacks. That was also what I thought we would get [from the dietitian].”* (Participant 10, North African origin, 32 years, diet-treated, first-time GDM)

Some women also expressed a need for dietitian-provided inspiration on healthier versions of traditional dishes, strategies for managing sweet cravings, and guidance on specific individual challenges such as hunger, lack of appetite, or unintended weight loss as part of the dietary therapy.

There were also unmet needs related to guidance on physical activity, and few participants recalled having in-depth discussions about incorporating physical activity into self-care with healthcare professionals, despite many expressing a need for more guidance on the topic.

Barriers to engaging in physical activity included challenges such as fitting exercise into busy schedules already filled with frequent medical appointments, family responsibilities (e.g., caring for young children), pregnancy-related fatigue, work commitments requiring time off, and long distances to exercise facilities. As one woman described:

*“I have appointments almost every week, so I’ve told my boss at work that I’ll be gone for about half a day every week because of this diabetes. She’s very understanding and kind, but it does mean that I’m away from work quite a lot to begin with, and then I need to pick up my son. That’s why I don’t exercise as much as I’d like to. Often, when he finally goes to sleep, I need to work again. And if I do get to exercise, it’s at 10 p.m. at the gym, which isn’t close to my house. So, it’s a bit hard to fit it all in.”* (Participant 12 of European origin, 37-years, insulin-treated, recurrent GDM)

Additional obstacles included a lack of information and uncertainty about suitable exercises, particularly for women dealing with pregnancy-related pain, discomfort, or limited physical activity prior to pregnancy. Some participants noted that they lacked information on safe and practical strategies for exercising during pregnancy while managing GDM:

*“Regarding exercise, I don’t think they say much. You’re told right from the beginning of pregnancy that you need to remember to exercise, but then everyone says you shouldn’t exercise or start anything new that you weren’t doing before. So, it’s like, okay, if you didn’t exercise before, then you might as well just sit down on the couch?”* (Participant 10, North African origin, 32 years, diet-treated, first-time GDM)

Common recommendations like ‘be active for 30 min’ or ‘take a walk after every main meal’ where often seen as too generic, lacking actionable guidance tailored to their current situation, or insufficiently motivating:

*“I would have liked there to be some advice on what we could do when the blood sugar was high, for example, or what we could do preventively without it being something like, ‘you need to go for a one-hour walk,’ because that’s not possible when you’re heavily pregnant.”* (Participant 8, Danish origin, 41 years, diet-treated only, first-time GDM)

Despite these challenges, most participants adapted their routines to include physical activity in ways that suited their circumstances. Common activities included post-meal walks, weekly swimming, yoga, and home-based exercises. These adjustments demonstrated their efforts to manage their condition while balancing other responsibilities and demonstrated their awareness of physical activity’s impact on BG levels:

*“I use steps, mostly 8,000–9,000 every day. […] Almost after every meal. In the morning about 30 minutes and, in the afternoon, also like 30 minutes mostly, and in the night just for 10 to 15 minutes. […] This regularly started after my diagnosis.”* (Participant 15, South Asian origin, 39 years, diet-treated, first-time GDM)

*“I quickly figured out that I could eat as I usually do if I go for a walk with the dog afterward or get some exercise—my blood sugar stays completely stable. So, it’s just important that as soon as I’ve eaten, I get out and exercise.”* (Participant 2, Danish origin, 33 years, insulin-treated, first-time GDM).

Some participants noted that maintaining regular physical activity was challenging due to fatigue, discomfort, and other pregnancy-related symptoms. Although they recognised the importance of being active for their health and their baby’s well-being, they did not detail specific strategies for consistently staying active.

### Theme 6 dietary challenges during transition to insulin therapy

Dietary challenges were particularly prevalent during the transition to insulin therapy. Requiring insulin introduced an endocrinologist as part of the women’s care team, and some women noted inconsistencies in dietary recommendations provided by the dietitian at diagnosis and the endocrinologists during the transition to insulin therapy, which caused confusion and frustration. These differences were particularly evident in approaches to carbohydrate management and the use of a smartphone app for supporting carbohydrate counting. Some women struggled to reconcile the diverging recommendations on their own, as the guidance was often unclear or contradictory:

*“What is a good balance? I find that hard to figure out. When they [endocrinologists] say, ‘Well, now it’s Christmas, so if you want to eat some cake, just take a bit more insulin.’ But how often is it okay to do that? Can I do it once a week, once a month, or once a day? […] I feel like I’m getting very different information”* (Participant 12, European origin, 37-years, insulin-treated, recurrent GDM)

In addition, participants described several practical challenges in managing insulin therapy and carbohydrate intake. These included lacking skills in basic carbohydrate counting, such as estimating carbohydrate amounts in foods, understanding carbohydrate portions sizes, and determining recommended daily intakes of carbohydrates, as well as integrating the use of a smartphone app or similar tools into their daily routines for guidance on this.

As part of their insulin therapy approach, endocrinologists systematically introduced a specific smartphone app—designed for individuals with diabetes—to help insulin-treated women with GDM estimate carbohydrate portion sizes more accurately for improving glycaemic control. However, many participants felt they would have benefited from earlier access to this app to support their dietary adjustments before requiring insulin. Additionally, these participants felt they lacked sufficient guidance on using the app and perceived that the endocrinologists assumed merely mentioning the app would be enough for them to adopt it effectively:

*“It would have been helpful if the diabetes doctor had said, ‘I recommend counting carbohydrates; you can use this app, and here’s how it works.’”* (Participant 9, Danish origin, 36 years, insulin-treated, first-time GDM).

Some also emphasised the need for more practical demonstrations and education on carbohydrate management and app usage provided by a dietitian:

*“The diabetes doctor told me I could have a certain number of grams of carbohydrates for snacks and the same for my main meals. [...] The dietitian could have explained this and provided a more detailed demonstration of how to use the app [for estimating carbohydrates].”* (Participant 19, Danish origin, 41 years, insulin-treated, recurrent GDM)

In general, some appreciated the app’s visual aids for estimating carbohydrate content, but others found the process of measuring foods overwhelming, especially when combined with multiple dietary restrictions:

*“It’s like, oh no, do I really have to start weighing and measuring everything? I already feel like there are so many restrictions.”* (Participant 6, Danish origin, 33 years, insulin-treated, first-time GDM)

The lack of clear guidance and practical education on recommended minimum and total carbohydrate intake—both overall and per meal—since diagnosis added to the challenges participants faced during pregnancy:

*“[…] I’m still unsure—should I eat 30 or 50 [grams of carbohydrates]? Is it the maximum, minimum, or average? I think it’s the minimum, but it would be nice having someone say, ‘This is how much rice you should eat’. […] Practical exercises and teaching about carbohydrate amounts would help. […] I think I’d feel more confident if I practiced measuring.”* (Participant 12, European origin, 37-years, insulin-treated, recurrent GDM)

Consequently, some women had completely avoided carbohydrates or misinterpreted the endocrinologist’s guidance, indicating uncertainty or misconceptions about whether the recommendations referred to the carbohydrate content in grams or the total weight of high-carbohydrate foods:

*“I weigh my food because the diabetes doctor said I should have 20–50, and that I could eat 20 grams of pasta or something similar.”* (Participant 14. North African origin, 34 years, insulin-treated, first-time GDM)

## Discussion

This study explored women’s experiences with healthcare and self-care in GDM within a Danish context, with a particular focus on dietary education and its delivery, as well as broader healthcare information and support needs. While several of our findings align with previous qualitative research highlighting women’s need for timely and personalised dietary guidance in GDM, this study extends existing knowledge by foregrounding how the delivery of dietary education shapes women’s ability to translate recommendations into everyday self-care. Rather than focusing primarily on the content of dietary recommendations or cultural tailoring, our findings demonstrate that gaps related to the timing and format of dietary education (group-based versus individual), the degree to which dietary guidance was concrete and actionable, diabetes-specific dietitian competencies, continuity, and opportunities for follow-up play a central role in shaping women’s self-care experiences. Although most women reported feeling generally supported by healthcare professionals, they described shortcomings in the timeliness, personalisation, emotional responsiveness, and consistency of dietary guidance. In particular, experiences with group-based dietary education that some women perceived as insufficiently tailored, combined with limited opportunities for timely individual clarification and follow-up, contributed to uncertainty and frustration in dietary self-care. In addition, we identify the transition to insulin therapy as a distinct period of vulnerability characterised by unmet educational needs, limited practical skills in carbohydrate management, and inconsistent guidance across healthcare professionals. By situating women’s experiences within a Danish healthcare context, this study highlights how organisational structures and interdisciplinary coordination shape access to dietary education and may mitigate or exacerbate inequities. Together, these findings underscore the need for earlier, more individualised, and better-coordinated dietary education, supported by interdisciplinary collaboration, to enhance confidence, reduce uncertainty, and strengthen self-care throughout pregnancy while ensuring consistent and concordant guidance across healthcare professionals.

Most women in our study highly valued the formal healthcare services received during pregnancy, describing healthcare professionals as thorough, supportive, and effective in addressing medical concerns. However, participants also expressed a strong need for more timely and responsive information immediately after diagnosis to better understand the condition, reduce emotional strain, and initiate necessary behavioural changes. In particular, delays in accessing dietetic guidance following diagnosis were perceived as challenging, as dietary self-care plays a key role in the initial management of GDM.

Dietary changes and demands placed on women following a GDM diagnosis may also have important implications for mental well-being both during and after pregnancy. Several participants described feelings of confusion, frustration, guilt, or uncertainty related to their condition or dietary recommendations, particularly in the period following diagnosis and during treatment transition. Unclear or overly generic guidance appeared to exacerbate these emotional responses, sometimes leading to anxiety around food choices, overly restrictive eating behaviours, or disengagement from dietary self-care recommendations. These findings of emotional burden are supported by evidence from a recent Danish study, in which up to one third of women with prior GDM reported self-blame or guilt related to prioritising their own health and concerns about their child’s future health [43].

While most women were able to identify reliable sources of health information and seek support, some reported relying heavily on informal sources, such as family members with health-related expertise, friends, or colleagues with previous GDM experience, or social media and other online sources. This reliance reflects gaps in the timeliness and accessibility of formal dietary support and highlights potential health inequalities, as not all women have access to such networks. It also increases the risk of misinformation when advice or self-care practices do not align with clinical recommendations for GDM. Previous research has similarly identified insufficient access to timely, reliable health information and uncertainty about trustworthy online resources as barriers to effective self-care in GDM [44–46].

Within the Danish healthcare system, dietary management of GDM is primarily delivered by hospital-based dietitians working across multiple medical specialties with competing clinical priorities. As a result, dietetic care for GDM is often provided on an ad-hoc basis. Variations in weekly referral volumes for women diagnosed with GDM, together with constraints in staffing and resource allocation, may further limit capacity and make timely access to dietetic consultations challenging. These organisational conditions suggest that delayed access to dietetic guidance following GDM diagnosis reflects broader structural challenges within the Danish healthcare system rather than being specific to the study site and therefore warrants attention at a system level. Against this backdrop, our findings underscore the need for universally accessible, trustworthy, and comprehensive healthcare information, alongside personalised guidance and timely access to relevant healthcare professionals. One participant suggested the development of a user-friendly healthcare-delivered digital platform offering practical dietary advice, recipes, and meal planning tools tailored to individual needs. Such an approach could provide structured, evidence-based guidance while reducing reliance on informal information sources and potentially improving cost-efficiency.

However, although digital tools, including web-based platforms and smartphone applications, show promise in supporting self-care in GDM, evidence regarding their effectiveness remains limited [47]. In our study, the introduction of an app for carbohydrate counting among insulin-treated women with GDM was challenging to use without comprehensive guidance. These challenges may be particularly pronounced for women with lower levels of health, language, or digital health literacy. Without adequate introduction, follow-up, and adaptation to individual needs, digital solutions risk increasing uncertainty or cognitive burden rather than facilitating self-care. Within this context, simple, low-threshold digital strategies—such as proactively providing newly diagnosed women with GDM links to reliable websites and short educational videos—may represent a feasible way to ensure timely access to high-quality information during a critical early phase of the care pathway, rather than replacing, personalised dietetic guidance and support.

The participants in our study did not express a single preferred method for receiving dietary education, underscoring the need for diverse educational formats that accommodate different learning styles and preferences. Both group sessions and individual sessions, including virtual options (e.g., telephone or video calls), were appreciated, with virtual dietitian follow-ups particularly favoured. These findings are consistent with previous research indicating that women with GDM prefer flexible, personalised dietary therapy approaches that address individual needs and provide tailored support [48]. Given the limited evidence on optimal dietitian-led care models in GDM—particularly regarding the ideal type and frequency of dietetic consultations—this remains an important area for further investigation [49–51].

A key finding of this study is the unmet need for diabetes-specific education to support effective GDM self-care. Women with previous GDM who had received care at other hospitals in past pregnancies reported substantial variation in the content of dietary education across hospitals, with some offering more diabetes-specific guidance than others. This unmet need was further compounded by inconsistencies between dietary recommendations provided by dietitians and those given by endocrinologists for insulin-treated women. Previous research has similarly identified the absence of structured carbohydrate counting and individualised carbohydrate portion planning as barriers to optimal dietary management in GDM [52]. Beyond dietary guidance, participants also described self-care recommendations related to physical activity as insufficiently diabetes-specific and tailored to their individual needs. In particular, the common recommendation to engage in 30 min of daily physical activity was perceived as overly generic and difficult to implement, especially for women unaccustomed to regular exercise or facing barriers such as time constraints, pregnancy-related fatigue, pain, or logistical challenges. The lack of individualised diabetes-specific guidance—particularly regarding safe exercise during pregnancy and practical strategies for integrating physical activity into daily routines—was a source of frustration and concern. Future interventions should therefore build on women’s pre-pregnancy physical activity habits, physical limitations, and expressed concerns, while also exploring alternatives to the standard recommendation of 30 min of daily activity or walking. Such tailored approaches may promote greater participation and more sustainable engagement in physical activity during pregnancy with GDM. Collectively, these findings highlight the need for more standardised yet personalised self-care education, strengthened interdisciplinary collaboration among dietitians, endocrinologists, and obstetricians, and the adoption of a unified nutrition and self-care behavioural approach to GDM across Danish hospitals—an approach that is currently lacking internationally [53]. Our findings are further supported by a recent qualitative systematic review of healthcare professionals’ perspectives on GDM care in high-income countries, which identified systemic barriers including limited multidisciplinary collaboration and a lack of consensus in practice guidelines [54]. Such structural challenges may contribute to variability in care delivery across hospitals, including differences in the scope and content of dietary and physical activity guidance offered to women with GDM.

Improved coordination among healthcare professionals would also ensure consistent dietary information throughout the care pathway, strengthening patient support in GDM self-care. Moreover, previous research has highlighted challenges related to inconsistent dietary recommendations [46] and emphasised the importance of a multidisciplinary approach to enhance resource access, leverage expertise, and reinforce critical information [55]. Furthermore, registered dietitians working in diabetes care should have advanced expertise in diabetes education and management to support optimal health-related outcomes. However, our findings indicate that this was not the case at the local hospital study site. In countries such as the United States, Canada, and Australia, registered dietitians are required to obtain and maintain advanced certification as diabetes educators, ensuring a high standard of diabetes care [56–58]. In contrast, current Danish clinical guidelines do not emphasise such specialisation, highlighting the need to restructure dietitian training programs in this area. Strengthening these competencies would support the delivery of standardised, high-quality, personalised, and practical dietary guidance—including carbohydrate management—ultimately enhancing dietary self-care and adherence among women with GDM.

Many women in our study expressed a preference for improved access to peer support, such as healthcare-facilitated group sessions. Previous studies have highlighted the value of these sessions in fostering experience-sharing, social connections, and a sense of community among women with GDM [28, 46]. Although research on peer support in women with GDM is limited, one qualitative study found that integrating peer counselling into a healthy eating and physical activity intervention enhanced engagement and motivation among women with a history of GDM [58]. In that study, participants reported that receiving emotional, appraisal, and informational support from both a peer counsellor and an exercise specialist was particularly valuable for adopting healthier behaviours [59]. This finding aligns with some of our participants’ views, although others were reluctant to participate in group education or peer support because they did not identify with other women with GDM—a finding that is also consistent with previous research [60]. Furthermore, a recent meta-analysis of health interventions based on Self-Determination Theory (SDT) concluded that social support and autonomous motivation are closely linked to positive behaviour change across diverse populations and contexts [61], suggesting that further exploration into the role of social support in GDM self-care is warranted. Notably, while dietary group sessions were frequently criticised for being impersonal and offering limited opportunities for individual engagement, group-based supervised exercise was generally regarded as beneficial and motivating, although logistical and scheduling constraints was perceived as a barrier for participation.

Previous studies have suggested that group-based educational sessions may be less effective for ethnic minority women with language barriers and limited health literacy, as these factors may increase the risk of miscommunication and hinder comprehension of key information [28]. However, our findings did not support this assumption. Although our participants represented a diverse group of women of non-Danish ethnic origin, we did not identify ethnicity-related differences in expressed needs or preferences regarding educational format or support, information provision, or peer support. This finding is unlikely to be explained by higher educational attainment or uniformly strong health literacy among women of non-Danish ethnic origin. Approximately half of these women had completed only primary school education. Moreover, most women of non-Danish ethnic women were able to communicate in Danish and had therefore received group-based dietary education rather than individual counselling. Despite this, we did not observe systematic differences in preferences or perceived support needs. In addition, several women of non-Danish origin—despite lower formal education—demonstrated strong functional health literacy through practical strategies, such as actively seeking information, drawing on informal social networks, and using available tools to support self-care. Together, these findings suggest that flexible and personalised approaches to care, rather than assumptions based on ethnicity or educational level alone, may be crucial for ensuring equitable access to information and support in GDM care.

Our study provides valuable insights into women’s experiences with GDM-related healthcare and self-care, particularly dietary education. A key strength of the study is the inclusion of diverse participants, encompassing both first-time and recurrent GDM cases, as well as women from varied ethnic backgrounds. Participants were also at different gestational stages and differed in treatment type, including women whose glucose levels were managed solely through dietary adjustments and those who required insulin therapy as an add-on. This diversity enhances the transferability of findings and provides a comprehensive understanding of the experiences and needs of women with GDM. Nevertheless, despite efforts to recruit a representative sample of women with GDM, the study population was relatively homogeneous. Participants predominantly came from traditional family structures, and many women from ethnic minority groups were Danish-speaking and employed. Furthermore, across ethnic backgrounds, participants generally demonstrated higher educational attainment compared to the overall Danish population.

## Conclusions

This study explored healthcare and self-care experiences among a diverse group of women with GDM, focusing on dietary education and how its delivery influences self-care. We identified several unmet needs and opportunities to enhance healthcare services and self-care support for women with GDM in Denmark. Our findings underscore the importance of timely, accessible, and trustworthy information, particularly immediately following diagnosis, as well as the need for more personalised, diabetes-specific education and ongoing dietary support, acknowledging that self-care demands in GDM, and women’s emotional well-being are closely interconnected.

Importantly, this study highlights how the delivery of dietary education—rather than content alone—shapes women’s ability to manage GDM, including the timing, format, continuity, and coordination of care. Periods of heightened vulnerability, such as the transition to insulin therapy, were characterised by unmet needs for more practical dietary skills and by inconsistent guidance across healthcare professionals. Women’s needs also encompassed tailored and motivational guidance on safe physical activity and, for some, access to peer support to strengthen self-care. At the same time, preferences regarding educational formats, peer interaction, and follow-up varied considerably, emphasising the importance of flexible, individualised approaches rather than one-size-fits-all solutions. Together, these insights highlight the need for earlier, more individualised, and better-coordinated dietary education within GDM care pathways. Addressing modifiable features of healthcare delivery—such as interdisciplinary coordination, continuity of support, and responsiveness to individual needs—may enhance self-care, reduce uncertainty, and ultimately improve health-related outcomes for women with GDM and their children.

## Supplementary Information

Supplementary Material 1. Interview guide.

## Data Availability

The data that support the study findings are held by Steno Diabetes Center Copenhagen (SDCC) and have not been made available to preserve the anonymity of participants and healthcare professionals involved in their care.

## Abbreviations

BE: Bettina Ewers (author)
COREQ: Consolidated criteria framework for reporting qualitative studies
ED: Emma Davidsen (author)
GDM: Gestational diabetes mellitus
KKN: Karoline Kragelund Nielsen (author)
MJH: Marianne Juhl Hansen (author)
SDCC: Steno Diabetes Center Copenhagen
