# Supplementary material for: Women’s experiences of navigating gestational diabetes in Denmark: a qualitative study of healthcare and self-care, with a focus on dietary education and delivery
Source: BMC Pregnancy Childbirth. 2026 Apr 16;26:570. doi: 10.1186/s12884-026-09055-8 (PMC13202973; doi:10.1186/s12884-026-09055-8)
Supplement: Supplementary file 1 — Supplementary Material 1. Interview guide. [file 12884_2026_9055_MOESM1_ESM.pdf]

Interview guide (English version)

Introduction

Before starting the interview, confirm that the participant has signed and returned the written informed consent.

Purpose: To create a safe, comfortable, and trusting interview setting and to ensure that the participant has understood all relevant information about the interview.

Method: Semi-structured qualitative interview, guided by a predetermined thematic framework.

<u>Prior to commencement of the interview and audio recording</u>

- Thank you for agreeing to participate in this interview.
- The interview will last up to 45 minutes.
- The purpose of this study is to explore women's experiences of managing gestational diabetes, with particular focus on self-care practices and support. Your perspectives will help us better understand interactions with healthcare services and everyday self-care and may inform improvements in future gestational diabetes care and support.
- This interview will also be part of research informing the development and testing of a programme aimed at improving self-care education at Steno Diabetes Center Copenhagen, in collaboration with the Department of Gynaecology and Obstetrics at Herlev Hospital, during the period 2023–2025. We are interested in learning from your experiences and perspectives.
- This interview is conducted in a confidential setting. All information you share will be treated confidentially and used only in anonymised form.
- Only the interviewer and two other members of the research team will have access to the interview data.
- Information from your interview will not be shared with your pregnancy doctors, midwife, or other healthcare professionals.
- The interview will be audio-recorded for research purposes. The recording will be anonymised, and only your first name will be used during the recording. Any quotations used in publications or presentations will be fully anonymised.
- The interview follows an interview guide with key topics and questions, but there are no right or wrong answers.
- You may choose not to answer any question.
- You may withdraw from the interview at any time without giving a reason.
- I may take notes during the interview to support the conversation.

Do you have any questions before we begin and before I start the audio recording?

Topic area	Questions
Demographics	Could you start by telling me a little about yourself?<br><br>• What is your first name and age?<br>• Are you currently in a relationship?<br>• Do you live alone or with others? (a spouse/partner)<br>• Do you have children?<br>• What is your highest level of education?<br>• Are you currently working, studying, or staying at home?<br>• Where were you born and raised? (Country/countries)<br>• What languages do you speak in your everyday life at home?<br>• What is your mother tongue (first language learned in your childhood)?<br>• Do you practise a religion?
Medical background	• When is your expected due date?<br>• At what point in your pregnancy (gestational week) were you diagnosed with gestational diabetes?<br>• Have you previously been diagnosed with gestational diabetes in an earlier pregnancy?
Understanding and experiences of GDM	***Personal experience***<br>• Can you describe what went through your mind when you found out that you had gestational diabetes?<br>• Have you reflected on why you think you developed gestational diabetes?<br>• Have you thought about whether there is anything you can do yourself to manage your gestational diabetes?<br><br>***Family and social context***<br>• Who in your family or social network knows that you have gestational diabetes?<br>• How did they react when you told them?<br>Do you feel that your relatives are concerned about your health during pregnancy?
Everyday life with GDM	• Has anything changed in your own or your family's everyday life since you were diagnosed?<br>• How do you experience your everyday life now that you have gestational diabetes? (Is it more difficult?)<br>• Are there things you miss being able to do as you did before? (E.g., physical limitations, psychological or emotional limitations or changes related to food and eating?)

Topic area	Questions
Experiences with healthcare and existing support	Next, I would like to hear about your experiences with healthcare professionals following your diagnosis of gestational diabetes (e.g. doctors, midwives, nurses, dietitians).<br><br>• What is it like for you to talk with healthcare professionals about your health? (CHAT red) E.g.:<br>○ Do you feel that you talk about what is important for you? (Why/why not?)<br>○ Do you feel able to ask questions and receive clear answers?<br>○ Do you feel able to use what you learn from these consultations in your everyday life? If yes, then how? If no, what do you think could be improved?<br><br>• Do you talk about your health with others besides health care professionals? (CHAT: supportive relations)<br>• How have you experienced using the information or support you have received from health professionals? (Do you understand the information? Is there anything you feel is missing?)<br>• Is physical activity something you talked or talk about with the health care professionals during your pregnancy and after your GDM diagnosis? (if yes: 1) with whom and 2) how have you used it?)<br>• Have you discussed physical activity with healthcare professionals during your pregnancy and after your GDM diagnosis? (If yes: with whom, and how have you used this advice?)<br>• Have you discussed diet or dietary recommendations with healthcare professionals during your pregnancy?<br>• How did your experience the waiting time between being diagnosed and your session with a dietitian?<br>• How did you experience your session with the dietitian after being diagnosed? (Was the dietitian session group-based or individual? Did you feel there was enough time? Was the session helpful? If applicable: What was it like to meet other women with GDM and talk about food?)<br>• Were you encouraged to count carbohydrates? (If so, by whom (doctor or dietitian)? How have you used carbohydrate counting in your daily life?)
Health knowledge and information sources	• Do you feel you that know enough about gestational diabetes or how to take care of your health?<br>○ If no, what would it mean for you to know more?<br>○ What would you like to know more about?<br>• Where do you usually obtain information about your health? Which sources or who do you trust the most and why? (CHAT) E.g., healthcare professionals, family or friends, the internet (websites, social media, forums, apps), books, magazines, religious communities, pharmacies, or others).

Topic area	Questions
Perceived need for health services	• What would be important for you if you wanted to change a health-related behaviour?<br>• What should a healthcare service include to help you live a good life during pregnancy with diabetes? E.g., support with 1) Eating habits; 2) Being more physically active; 3) Measuring your blood sugar; 4) Remembering to take your medication/insulin; or 5) A combination of these?<br>• Is it a challenge for you to have several visits in the health care system during your pregnancy?<br>• Do you find it challenging to attend several healthcare appointments during pregnancy? (Where do you feel your limit is? Would you be willing to attend several appointments with a dietitian or physiotherapist? Would this be easier if appointments were coordinated with medical consultations?<br>• How would you feel about meeting other women with gestational diabetes in group-based sessions?<br>• How would you feel about being in a group with women from similar or different backgrounds?<br>• What kinds of services or support would motivate you to engage in health-promoting behaviours? (Use supplementary visual materials with examples must be used here)
Health perception and health behaviour	I have a few final questions about how you perceive your health and how you are coping now.<br>***Perception of health and well-being:***<br>• Can you name five things that you associate with a healthy life?<br>• When you think about how you take care of your health and well-being, what do you feel you are managing well?<br>• What do you do in your everyday life to take care of your health? (E.g., eat healthily, exercise, measure blood sugar, take your prescribed medication (insulin))<br>Which aspects of taking care of your health do you find most difficult to maintain?<br>***Eating habits (in detail)***<br>• What does healthy eating mean to you?<br>• Have your eating habits changed since you were diagnosed with gestational diabetes? (If yes, how)<br>• Who is primarily responsible for cooking in your household?<br>• Do you experience any barriers to eating in a healthy way? (E.g., cost, lack of time, lack of knowledge or support, family preferences or considerations, or other factors)<br>• On a scale from 1 to 10, how important is it to you that you and your family eat healthily?<br>***Exercise habits (in detail)***<br>• Before your pregnancy and GDM diagnosis, how physically active were you on a typical day?<br>• Health authorities recommend that pregnant women engage in at least 30 minutes of moderate-intensity physical activity per day. Do you think this recommendation is realistic for you?<br>Do you experience any barriers to being physically active? (E.g., pain, fatigue, distance to facilities, cost, lack of time, lack of knowledge, family responsibilities, weather conditions, or other factors?

Topic area	Questions
**End of interview**	• Is there anything else you would like to add, either now or after the recording has ended?<br>• Do you have any questions for me?<br>• Would it be acceptable for us to contact you again if we need clarification or elaboration on something you have said?<br>• Thank you very much for your time and for sharing your experiences.

The Conversational Health Literacy Assessment Tool (CHAT) is a tool focusing on health competencies, developed by the Dept. of Public Health, Aarhus University.
